# Effect of portfolio assessment on student learning in prenatal training for midwives

**DOI:** 10.3352/jeehp.2011.8.2

**Published:** 2011-03-25

**Authors:** Nourossadat Kariman, Farnoosh Moafi

**Affiliations:** Department of Midwifery, Shaheed Beheshti University of Medical Sciences, Tehran, Iran.

**Keywords:** Evaluation, Portfolio, Learning, Satisfaction

## Abstract

The tendency to use portfolios for evaluation has been developed with the aim of optimizing the culture of assessment. The present study was carried out to determine the effect of using portfolios as an evaluation method on midwifery students' learning and satisfaction in prenatal practical training. In this prospective cohort study, all midwifery students in semester four (n=40), were randomly allocated to portfolio and routine evaluation groups. Based on their educational goals, the portfolio groups prepared packages which consisted of a complete report of the history, physical examinations, and methods of patient management (as evaluated by a checklist) for women who visited a prenatal clinic. During the last day of their course, a posttest, clinical exam, and student satisfaction form were completed. The two groups' mean age, mean pretest scores, and their prerequisite course that they should have taken in the previous semester were similar. The mean difference in the pre and post test scores for the two groups' knowledge and comprehension levels did not differ significantly (*P*>0.05). The average scores on questions in Bloom's taxonomy 2 and 3 of the portfolio group were significantly greater than those of the routine evaluation group (*P*=0.002, *P*=0.03, respectively). The mean of the two groups' clinical exam scores was significantly different. The portfolio group's mean scores on generating diagnostic and therapeutic solutions and the ability to apply theory in practice were higher than those of the routine group. Overall, students' satisfaction scores in the two evaluation methods were relatively similar. Portfolio evaluation provides the opportunity for more learning by increasing the student's participation in the learning process and helping them to apply theory in practice.

## INTRODUCTION

In recent decades, numerous innovations have occurred in the field of educational theory and practice. Traditional teacher-centered educational methods have been replaced with student-centered methods. Parallel with this trend, the evaluation system has also been changed from methods that evaluate knowledge to those that evaluate ability and competence. A wide variety of evaluation methods have been developed in this context [1]. A portfolio is a planned and purposeful collection of all kinds of documents that gives an impression of how tasks are fulfilled and how competence is developed [2, 3]. Interest in use of portfolios for assessment in the health-care professions has developed as a part of the move away from traditional testing towards the use of broader assessment approaches. This method provides a closer link between learning and assessment. The use of portfolios improves learning outcomes through the provision of feedback and attempts to assess students in areas difficult to assess by traditional methods (attitudes and personal attributes) [4, 5]. Portfolio collection requires the joint efforts of teacher and student in taking decisions, developing the portfolio's content, and establishing its assessment criteria [6]. Portfolios may include case-reports, a list of methods used, video tapes from consultations carried out, explanations of learning experiences in the clinical environment, reports on books or journals and report on research projects [7-10]. Portfolio advantages are as follows: it can assess areas that are difficult to test traditionally, attitude can be assessed, curriculum outcomes can be emphasized, it reflects a student-centered approach to learning, it helps to create critical thinking, and the breadth as well as depth of students' learning can be assessed [3, 4, 10, 11]. This method makes innumerable contributions to such issues as providing practice with equipment, being flexible, identifying the positive and negative environmental factors influential in the learning process, encouraging students' willingness to participate in activities, and orienting the activities accordingly [12, 13]. Several studies have shown that this method promotes learning [14, 15]. On the other hand, in some educational texts and articles in this area, lack of sufficient research in portfolios' reliability and validity for undergraduate students' assessment has been suggested as one of the weaknesses of this method [7, 8, 10]. The introduction of course work using portfolios as an assessment tool is rather new to educational research in Iran. Due to the lack of studies in this field in our country, the present study was performed to determine the effect of the portfolio evaluation method on learning and satisfaction of midwifery students of Shaheed Beheshti Medical University in prenatal practical training in 2008-09.

## MATERIALS AND METHODS

In this prospective study, all of the fourth semester midwifery students of two courses (n=40) were studied for 4 semesters from their first academic semester of 2007-08 to their second semester of 2008-09. Using the portfolio evaluation method in the apprenticeship and training of midwifery students was approved by the Education Development Organization of the nursing-midwifery faculty of Shaheed Beheshti Medical University and the Department of Midwifery. Student groups were allocated to the portfolio and routine evaluation groups by a simple random sampling method. Each group was composed of 20 students and there was no attrition during the research period. The pre-test was taken on the first day of training. Both groups were then trained by a midwifery instructor in a prenatal clinic. Finally, a post-test was administered on the last day of the training course. These tests contained 40 questions, which included 16 multiple choice questions (MCQs) on knowledge and comprehension levels. There were also two cases, with 16 MCQs for the first case focusing on the application and analysis levels, as well as two open-ended questions about the second case in which the students were asked to judge the management of a diagnosis suggested by the exam. The questions content validity was verified by the Shahid Beheshti Midwifery faculties.

The practical exams were carried out for the routine group based on practical training routine method evaluation on the last day of the training course. The portfolio group prepared their package based on the educational goals after being introduced to the portfolio evaluation method, performance of making the portfolio, portfolio contents, and a portfolio evaluation checklist. Their portfolios consisted of five complete reports on women who visited the prenatal clinic during the first nine days of the course, including for each, a history, physical exam, and method of patient management. The different groups of five women were selected by each student based on educational goals and the midwifery students' job description. The portfolio evaluation checklist utilized in our study was developed and first used by Davis et al. [16]. In this study, the validity of the checklist was calculated through content validity and its reliability was controlled again through the Kuder-Richardson test (coefficient KR=0.81). The checklist included six criteria; each criterion had minimum and maximum scores of 1 and 4, respectively.

The checklist's criteria included the following: portfolio content presentation, discussion about the portfolio, patient management, the ability to apply theory in clinical practice, use of educational resources, and scientific behavior (student learning from dealing with patients). During the last week of the training, the portfolios were presented and evaluated with the portfolio evaluation checklist while individual students were present with the teacher at the oral exam session. During the last day of their course, the post-test and clinical exams were administered to both groups. Students' satisfaction was assessed through the evaluation form. It included five phrases with five alternatives based on a Likert scale that was presented as follows: 1 for "completely dissatisfied," 2 for "dissatisfied," 3 for "no comment," 4 for "satisfied," and 5 for completely satisfied. The post-test and clinical exams were taken by routine evaluation methods without any intervention and the test scores were compared at the end of the next semester. This was carried out to determine the maintenance of evaluation method effects in prenatal practical training.

The data analysis was performed using SPSS ver. 15.0 (SPSS Inc., Chicago, IL, USA). The differences between pre-test and post-test scores were conducted by a paired sample *t*-test. The differences between the two groups were determined by *t*-test for numerical variables with a normal distribution. Moreover, the following methods were also utilized: a chi-square test for qualitative variables and a Mann-Whitney test for ordinal variables. Also, numerical variables with a non-normal distribution were employed and *P*<0.05 was chosen as the level of significance.

## RESULTS

As mentioned, two groups were considered. Both groups had similar mean ages, pretest scores, prerequisite course grades that they should have taken in the previous semester. The mean±SD of the ages of the portfolio group members was 20.25±0.55 while the routine evaluation group were similar, at 20.10±0.30. These characteristics were not statistically different (*P*>0.05). Fifty five % of the portfolio group students and 60% of the routine group students lived in the dormitory; the χ^2^ statistic did not show a significant difference between the two groups (*P*>0.05). Also, the *t*-test did not show a significant difference between the portfolio and routine groups' pre-test scores, which were 8.97±1.54 and 9.55±1.84, respectively (*P*>0.05). The portfolio group's scores on prerequisites lessons i.e., the pregnancy and child bearing theory, had a mean±SD of 16.30±2.10. This was not significantly different from the routine group (16.70±1.89).

The post-test scores are presented in Table 1 for both groups. It is noted that 11 portfolio group students (55%) and 6 (30%) routine group students had post-test scores of between 15 to 20 out of a total of 20. The mean post-test score of the portfolio group was 15.10±1.35, which was greater than the routine group (13.87±1.57). A *t*-test showed a difference between the two groups (*P*=0.01). The post-test scores were classified according to the questions' taxonomy as provided in Table 2. These classifications were taxonomy 1, knowledge and comprehension level questions; taxonomy 2, application, analysis, and synthesis level questions; taxonomy 3, evaluation and judgment level questions. There were not any statistically significant differences in the mean knowledge and comprehension level question scores on post-test between the two group (*P*>0.05). However, the mean scores on questions 2 and 3 in the taxonomy were higher for the portfolio group than the routine evaluation group.

**Fig. 1:**
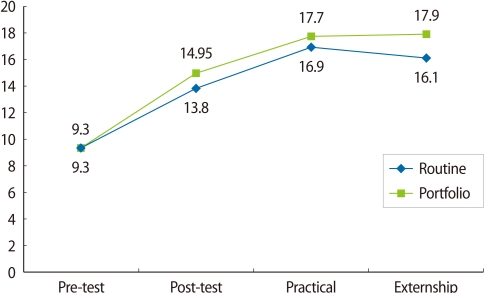
Pre-test, post-test, practical test, and prenatal externship test scores in the two groups.

**Fig. 2:**
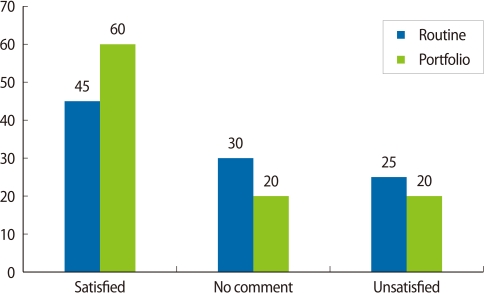
Students' satisfaction with the portfolio and routine evaluation methods.

The practical test score in the portfolio group was greater than that of the routine evaluation group (17.70±1.02 and 16.94±0.84, respectively). A *t*-test revealed that the difference was statistically significant (*P*=0.01). The mean scores of the two groups are presented in Fig. 1 for the pretest, posttest, practical and prenatal externship test scores. Moreover, the test scores were higher in the portfolio group than the routine group for the post, practical, and prenatal externship. Table 3 presents the frequency distribution of diagnostic and therapeutic management and communication between the theoretical and clinical leanings in the clinical exam. 10 students in the portfolio group (50%) and 5 students in routine group (25%) suggested at least one acceptable method of patient management and they were able to defend it. The difference between the two groups was statistically significant using Fisher's exact test (*P*<0.001). The 14 students of the portfolio group (60%) and 9 students (45%) of the routine group were satisfied (i.e., completely satisfied or satisfied) with the evaluation method. The Mann-Whitney test did not show a statistical difference between the two groups (*P*=0.13) as it is shown in Fig. 2.

## DISCUSSION

This study indicated that the portfolio evaluation method increases the students' learning level in prenatal practical training. Sahu et al. [3] performed a study in the Jawaharlal Institute of Postgraduate Medical Education and Research. They showed that students' learning and ability of self-assessment increased significantly for the portfolio group. Likewise, Tasdemir et al. [4] showed that the group for which portfolio evaluation along with cooperative learning was applied became more successful in passing the course than the other groups. Lambdin and Walker [17] concluded that the portfolio evaluation method increases self-assessment ability in students and makes them more independent in comparison to their classmates. In this research, scores on higher cognitive level question (analysis and judgment) were significantly greater for the portfolio group than the routine evaluation group. Scores for knowledge and comprehension did not show a significant difference between the two groups. The reason for this might be due to the fact that learning at these levels, i.e., knowledge and comprehension, is mainly acquired in theoretical classes. Thus, the portfolio method did not have any significant influence on the learning scores in comparison with the routine group. It should be noted that the portfolio evaluation method requires more time and sufficient training to involve teachers. Therefore, the routine method is more beneficial when the goal of training is for students to acquire knowledge and comprehension. On the other hand, the portfolio is more suitable if the goals to be achieved include higher cognitive skills. There are many important factors which determine whether to use the portfolio method. The most important one is to match this method with educational goals and course contents [2, 18]. This evaluation method provides more reliable results by taking information and assessing students from different perspectives during the learning process [19]. Portfolios provide more information about teacher and student activities. This information would be an appropriate source for the educational planning of each group by identifying the strengths and weaknesses of students [13, 16, 20]. Apple believes portfolios make an important contribution to providing information, evaluating facilities, having flexible goals, making educational plans, and identifying negative as well as positive environmental factors in the student evaluation process [12]. Using portfolios, teachers' biases are lowered [21, 22]. In the present study, student members of each group were already selected by the midwifery committee of the university. Thus, the selection process was not carried out randomly. To prevent this from affecting the quality of the study design, groups of students were randomly allocated into the two groups: portfolio and routine. Furthermore, the pre-test scores were not significantly different between these two groups (*P*>0.05).

Portfolio evaluation expands learning opportunities by increasing the students' participation in the learning process and helping them to apply theory in practice. Moreover, portfolios help students to develop three basic self-directed learning skills, assessing the quality of their own performance, formulating learning needs, and selecting future learning tasks.

## Figures and Tables

**Table 1:**
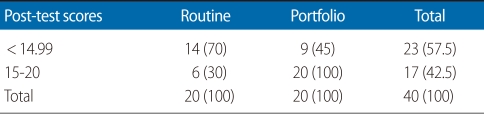
Post-test scores of prenatal practical training in the two groups

Values are presented as number (%).

**Table 2:**
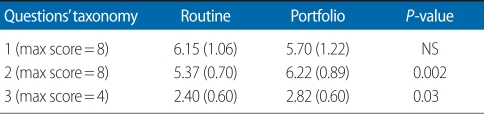
Pre-test scores according to the taxonomy questions in the two groups

Values are presented as mean (standard deviation).

**Table 3:**
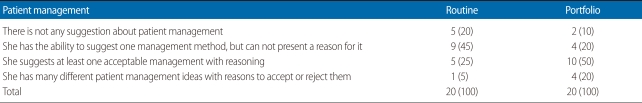
Patient management in the prenatal practical training in the two groups

Values are presented as number (%).
